# Lecture on Abrotanum

**Published:** 1885-02

**Authors:** J. T. Kent

**Affiliations:** St. Louis


### LECTURE ON ABROTANUM.

PROFESSOR J. T. KENT, M. D., ST. LOUIS.

A very useful remedy in marasmus or wasting. The tissues of the body have been wasting away. The child is hungry, but still wasting (Baryta carb., IODINE, and Natrum Mur.). The child is cross, depressed, and very peevish (symptoms which are common to marasmus). The face is wrinkled, as if old (Arg-n., Baryta carb., Sulph.). You feel the face, and it is cold and dry. There is general emaciation. (If only around the neck, while the body remains fat and plump—Natrum mur. Abrotanum does not produce this state and cannot cure it.) Ravenous appetite, all the while emaciating (*Iodine*, Natrum mur.). Living well, but growing thin (Natrum mur. and Baryta carb.). We find hard lumps in different parts of the abdomen—this is in marasmus. (Another remedy is Calcarea, but it has a liquid stool of curdled milk, undigested. Baryta carb. has these lumps but produces this condition only as a secondary symptom.) We have a distended abdomen; belly stands away out (also Calcarea, but the belly is higher up and like an inverted saucer. Baryta carb. has a large belly, but looks old). This remedy has alternate diarrhœa and constipation. Great emaciation of legs is prominent. Among the sensations, which are applicable to a great many diseases, are burning, hanging and swinging, gnawing, darting, tearing, cutting, rawness and dryness. In neuralgic rheumatism it is a very prominent remedy. Some remedies we are able to distinguish from all others by their mental state, others by their symptoms upon other parts of the body. This remedy has no very important mental symptoms. Anxious and depressed (Ars., Aconite, and Rhus.). This symptom occurs in neuralgia of the heart and stomach which is of a rheumatic kind, and comes on from change of disease or metastasis; useful in chlorosis, skin yellowish-green. Darting and tearing in carious teeth. Gnawing hunger is characteristic when indicated in any disease. Pains—cutting, gnawing, and burning are the most characteristic. (Arsenic has great burning.) Now there is a symptom which belongs to this remedy, and to no other, which is, a suddenly checked diarrhœa is followed by rheumatism. Stitching in the muscles *Abrotanum* will cure in six to twenty-four hours. Again, when an ulcer has been dried up, or when a hemorrhage has been checked, or when piles have been suppressed and evil results follow, think of *Abrotanum*.

In metastasis of rheumatism from the knee to the heart; in connection with this rheumatism there is a dry cough, which is a prominent symptom. A patient had a diarrhœa, which was checked; then rheumatism and a troublesome cough came on. *Abrot.* cured the rheumatism; the cough remained, which, however, passed off with the pathogenetic effects of the drug. Nosebleed in children—boys (also Ferrum-phos.). In girls fourteen or fifteen years of age, who have suppression of the menses followed by nosebleed, this is a remedy (also Bry.). If the pains fly all over the body, Puls.; but if the disease make one grand move, as from diarrhœa to rheumatism, *Abrotanum.* Eruptions come out on the face, are suppressed, and the skin becomes purplish. After scarlet fever, hydrocele (Apis and Puls.). After Aconite and Bryonia it has cured pleurisy—hence we see its action on the serous membranes. It prefers the left side. Burning, darting, and tearing pains in the left ovary were cured by Abrotanum after Apis had failed.

